# Effect of Homologous and Heterologous Booster in COVID-19 Vaccination

**DOI:** 10.3390/ph17121734

**Published:** 2024-12-22

**Authors:** Marija Vukčević, Mateja Despot, Aleksandra Nikolić-Kokić, Duško Blagojević, Milan Nikolić, Ana Banko, Tanja Jovanović, Dragana Despot

**Affiliations:** 1 Institute for Biocides and Medical Ecology, Trebevićka 16, 11030 Belgrade, Serbia; marija.vukcevic@biocidi.org.rs (M.V.); drdespot@hotmail.com (D.D.); 2 Faculty of Medicine, University of Belgrade, dr Subotića 8, 11000 Belgrade, Serbia; drdespotmateja@gmail.com (M.D.); ana.banko@med.bg.ac.rs (A.B.); 3 Department of Physiology, Institute for Biological Research “Siniša Stanković”—National Institute of Republic of Serbia, University of Belgrade, Despota Stefana 142, 11000 Belgrade, Serbia; dblagoje@ibiss.bg.ac.rs; 4 Faculty of Chemistry, Department of Biochemistry, University of Belgrade, Studentski trg 12-16, 11158 Belgrade, Serbia; mnikolic.chem@gmail.com

**Keywords:** COVID-19, Pfizer, Sinopharm, Sputnik vaccine, anti-S1 IgG antibodies

## Abstract

**Background:** COVID-19 became a global health crisis in early 2020, and the way out of the crisis was the rapid development of vaccines by Sinopharm, Pfizer, and Sputnik, among others, which played a crucial role in controlling the pandemic. Therefore, this study aims to investigate the long-term immune response by measuring the levels of anti-S1 IgG antibodies induced by homologous and heterologous vaccination regimens. **Methods:** We investigated the titer of the anti-S1 IgG antibody produced for the viral surface antigen 3, 6 months after the second dose, before the third dose, and 1, 3, and 6 months after the third dose. **Results:** Anti-S1 IgG antibody levels significantly increased three/six months after the second dose and following the booster in individuals without prior COVID-19 infection who received all three homologous vaccine doses. The group that initially responded poorly to Sinopharm showed a significant and sustained increase after receiving the Pfizer booster. Additionally, prior SARS-CoV-2 infection between primary and booster vaccination boosted anti-S1 antibody titers in all individuals, regardless of the vaccine used. The highest vaccine efficacy was observed during the primary vaccination period and declined over time, especially during the omicron-dominant period. **Conclusions:** The results suggest that while homologous and heterologous booster doses can significantly enhance anti-S1 IgG antibody levels, prior SARS-CoV-2 infection and the type of vaccine administered influence the duration and magnitude of the immune response.

## 1. Introduction

The emergence of COVID-19 became a global health problem around the world in early 2020 [1]. It has affected all people’s physical, social, and psychological well-being, especially healthcare professionals [2]. The disease was declared a pandemic by the WHO on 11 March 2020 [3]. The SARS-CoV-2 virus continues circulating among humans and constantly changes through mutations, with new variants occasionally emerging. These variants may have different characteristics that affect the spread of the virus or the effectiveness of existing vaccines and therapies. Recent studies and reports indicate that Omicron and its subvariants continue to dominate as the primary SARS-CoV-2 variants worldwide [4]. Characterized by over 30 mutations in the spike protein, Omicron represents the most divergent variant compared to the original strain. It exhibits higher transmissibility, partial immune evasion from vaccines or prior infections, and typically results in milder symptoms. However, it still poses significant risks to vulnerable populations [5]. The rapid development of vaccines has been the most important key to successfully controlling the pandemic [6]. Various types of vaccines based on different platforms (inactivated, vector-based, mRNA, and subunit vaccines) have been developed for immunization. The citizens of Serbia most frequently requested immunization with the inactivated Sinopharm vaccine for their primary vaccination [7]. Vaccines based on Pfizer’s novel mRNA technology and the vector-based Sputnik V vaccine were the second and third most popular choices [8]. Utilizing the oldest production platform, Sinopharm’s inactivated vaccine was developed in a short period. It contains the whole inactivated particles and adjuvant. The advantages of inactivated vaccines lie in their safety and ability to elicit a good humoral immune response. The immune response is triggered to all viral antigens, but only after multiple introductions and in the presence of an adjuvant [9]. The recommended plan for basic immunization with the Sinopharm vaccine was two doses with a four-week interval between doses.

After basic immunization with two vaccine doses, the immune response to SARS-CoV-2 wanes over time, making a booster dose necessary [10,11,12]. The waning of the humoral immune response after the initial vaccination, with reduced protection, was the reason WHO recommended the booster dose, especially for immunocompromised individuals and those with chronic disease. Our previous results have shown that the third dose of the Sinopharm vaccine as a booster vaccination causes a significant increase in antibody response when administered six months later [13].

Our work hypothesized how homologous and heterologous booster vaccinations enhance the declining humoral immune response triggered by the first SARS-CoV-2 vaccination, with antibody titers differing according to vaccine type and the occurrence of natural infection. Therefore, we investigated the quality of the humoral immune response previously elicited by two doses of a SARS-CoV-2 vaccine by measuring the titer of antibodies specific to the viral surface antigen (S1) while also investigating the diminished humoral immune response following both the primary and booster doses patients, mainly focusing on those who experienced natural infection either before or after the booster dose.

## 2. Results

**Figure 1:**
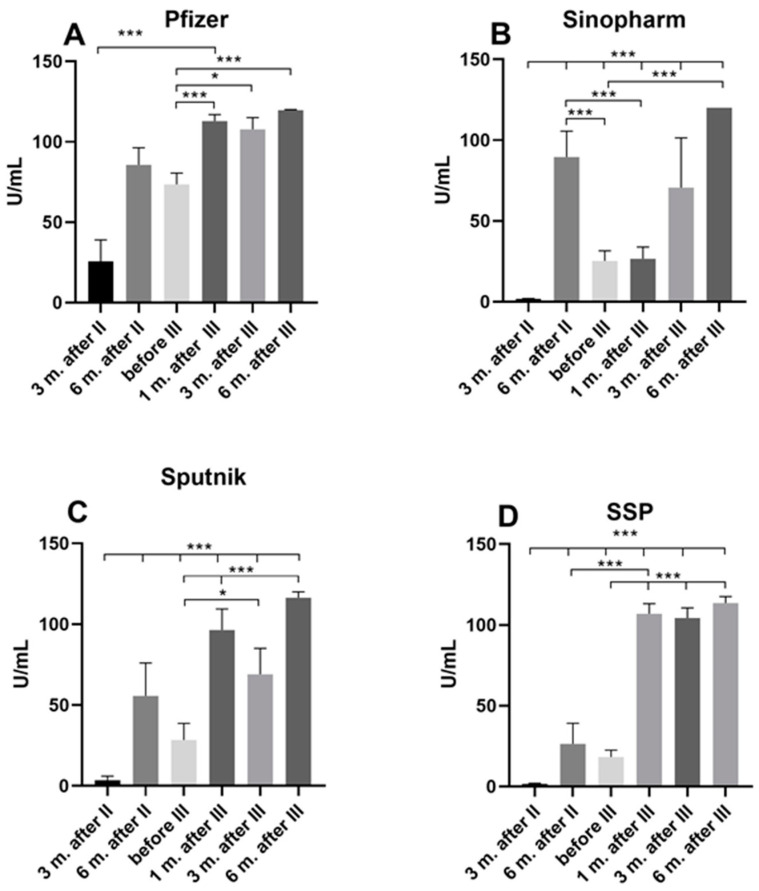
Marker of humoral immune response, anti-S1 IgG antibodies, after vaccination with (**A**) three (I–III) doses of Pfizer; (**B**) three (I–III) doses of Sinopharm; (**C**) all three (I–III) doses of Sputnik vaccine; and (**D**) a combination of Sinopharm and Pfizer vaccines (two doses of Sinopharm and third dose of Pfizer, SSP) in individuals with no history of COVID-19. * *p* < 0.05; *** *p* < 0.001.

The humoral immune responses, anti-S1 IgG antibodies in individuals vaccinated with one of three different vaccines (Pfizer, Sinopharm, and Sputnik), and the composition of the first two Sinopharm vaccines and the third Pfizer vaccine at 3 and 6 months after the second dose; before the third dose; and 1, 3, and 6 months after the third dose in 410 individuals (178 men and 232 women-Table 1) showed different profiles (Figure 1). We divided the subjects into one group with no history of COVID-19 during the observation period and the other group with a history of COVID-19 during the vaccination period (Table 2).

In the group of participants who received three doses of the Pfizer vaccine and had no history of COVID-19 during the observation period, anti-S1 IgG antibody levels showed an insignificant increase 6 months after the second dose, then a non-significant decrease before the third dose. A statistically significant increase in anti-S1 IgG antibody levels was detected 1, 3, and 6 months after the third dose (Figure 1A).

Anti-S1 IgG antibody levels in participants who received three doses of the Sinopharm vaccine were barely detectible 3 months after the second dose. The levels of anti-S1 IgG showed a statistically significant increase 6 months after the second dose compared to 3 months after the second dose and a statistically significant decrease before the third dose. This level remained approximately the same one month after the third dose and only reached a statistically significant increase 6 months after the third dose (*p* < 0.001) (Figure 1B).

The situation with the Sputnik vaccine was similar to that of the Sinopharm vaccine; the values were very low in the third month after the second dose. After 6 months, there was an insignificant increase and then an insignificant decrease before the third dose was administered. After administering the third dose of the vaccine, there was a statistically significant increase in anti-S1 IgG antibodies after the first month (*p* < 0.001). After 3 months, the level of anti-S1 IgG antibodies was still high, with a *p*-value < 0.05, and after 6 months, it was even higher, *p* < 0.001 (Figure 1C).

The fourth group of subjects received the first two doses of the Sinopharm vaccine, but as they did not show a satisfactory immune response, they received the Pfizer vaccine as the third dose. The levels of anti-S1 IgG antibodies were also very low 3 months after the second dose, as in the second group, which slightly increased after 6 months and decreased before the third dose. The values for anti-S1 IgG antibodies increased with high statistical significance immediately after the administration of Pfizer as a third dose in the first month and maintained these levels 3 and 6 months after the third dose (*p* < 0.001) (Figure 1D).

**Figure 2:**
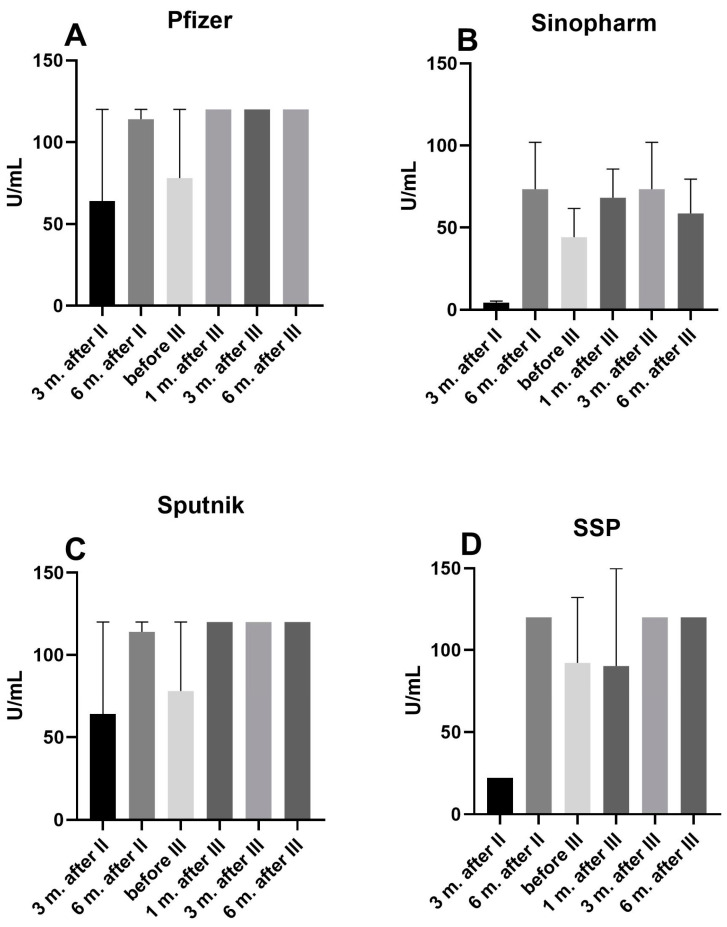
Marker of humoral immune response, anti-S1 IgG antibodies, after vaccination with (**A**) three (I–III) doses of Pfizer; (**B**) all three (I–III) doses of Sinopharm; (**C**) three (I–III) doses of Sputnik vaccine; and (**D**) a combination Sinopharm and Pfizer vaccines (two doses of Sinopharm and third dose of Pfizer, SSP) in individuals with a history of COVID-19.

The situation with anti-S1 IgG antibody levels in people who have received various vaccines but had COVID-19 differs from those who had no history of COVID-19. In the first (Pfizer), third (Sputnik), and fourth groups (Sinopharm and Pfizer combined), the results were almost identical: there was an increase 6 months after the second dose, and this value was maintained throughout the follow-up period, but without statistical significance (Figure 2A,C,D). In the second group, which received three doses of the Sinopharm vaccine, the situation was slightly different regarding the magnitude of the response at 6 months after the third dose (Figure 2B).

**Figure 3:**
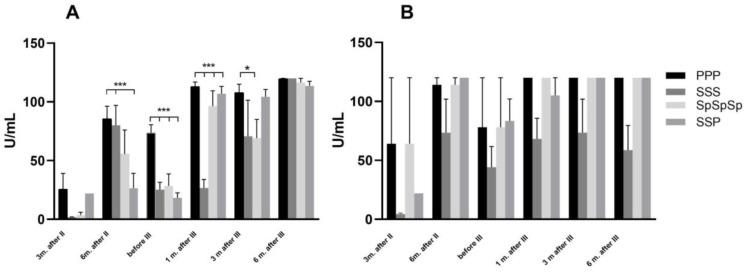
Comparison of different vaccines in time points 3 and 6 months after the second (II) dose; before the third (III) dose; and 1, 3, and 6 months after the third dose. Marker of humoral immune response, anti-S1 IgG antibody, during vaccination with all three doses of Pfizer (PPP); all three doses of Sinopharm (SSS); all three doses of Sputnik vaccine (SpSpSp) and a combination of Sinopharm and Pfizer vaccine (two doses of Sinopharm and third dose of Pfizer, SSP) in individuals without (**A**) and with (**B**) a history of COVID-19. * *p* < 0.05; *** *p* < 0.001.There were no differences among measured time points between groups in subjects with COVID-19 (ANOVA showed no statistical significance among groups) (Figure 3B). In addition, subjects who had COVID-19 had higher baseline values 3 months after the second dose in all groups studied, but again without statistical significance. However, subjects who had received three doses of the Sinopharm vaccine and had a history of COVID-19 had higher antibodies one month after the third dose (*p* < 0.05). However, the situation reversed after 6 months (Figure 4). In addition, the history of COVID-19 had prolonged the immune response in the SSP group up to 3 months after the second dose (*p* < 0.05), a difference that was similar before the third dose (*p* < 0.001).

When we compare the different vaccines (Pfizer, Sinopharm, Sputnik, and Pfizer–Sinopharm combination) at the same observation times 3 and 6 months after the second dose, before the third and one month, 3 and 6 months after the third dose in the subgroups that did not have COVID-19, analysis of the differences in anti-S1 antibody levels showed that participants who received SSP had statistically lower levels of anti-S1 IgG antibodies 6 months after the second dose than the groups receiving PPP and SSS (*p* < 0.001) (Figure 3A). In the group that received all three Pfizer vaccines, levels of anti-S1 IgG antibodies remained higher before receiving the third dose and differed significantly from the other three observed groups (*p* < 0.001). The levels of anti-S1 IgG antibodies one month after the third dose show that the group that received all three doses of Sinopharm had statistically lower antibody levels than the other groups, which persisted after three months compared to Pfizer (*p* < 0.05). Three months after the third dose, Sputnik had lower values than Pfizer (*p* < 0.05). Six months after the third dose, all vaccines have similar maximum values of antibodies without significant differences (Figure 3A).

The statistical analysis showed no impact of age, gender, or accompanying illnesses on the humoral immune response. Data are not presented.

## 3. Discussion

Several factors influence vaccine effectiveness, including host-related factors such as age, underlying medical conditions, and prior infection history, as well as pathogen-related factors like circulating virus variants and vaccine-related aspects such as the type of vaccine and time since the vaccination. Immunization against COVID-19 in Serbia began in January 2021. As various vaccines were available, citizens could choose which vaccine they wanted to receive. The subjects included in this study were primarily vaccinated with the Sinopharm, Pfizer, or Sputnik V vaccines. All these vaccines were produced for the original Wuhan variant of the virus.

Based on the genome sequencing analysis of viruses isolated from nasopharyngeal swabs of infected patients in Serbia, three epidemiological waves of COVID-19 were observed in the period from March 2020 to March 2022 when the study was conducted: the dominance of the Alpha variant in the period from late December 2020 to April 2021; the Delta variant from July 2021 to 31 December 2021; and the Omicron variant from 31 December 2021 to March 2022 [13]. These data allowed us to monitor the efficacy of the vaccines in relation to the Delta and Omicron variants. Based on the date of the last positive PCR results, the participants were divided into two groups according to their history of SARS-CoV-2 infection: a group of patients who had the infection before the buster vaccination and a group of patients who had the infection after the booster dose. All subjects who had positive PCR results before the booster vaccination were infected with the alpha variant, and most of the subjects infected after the booster vaccination who had symptoms of COVID-19 became ill during the period when the delta and omicron variants were prevalent.

The immune response to the vaccine usually peaks 14–28 days after the second dose of the first series [13,14,15]. In contrast to antibodies of the IgG classes against many pathogens, which persist forever, antibodies against SARS-CoV-2 decrease slowly and fall below the protective level several months after vaccination [11,13,15,16]. According to the literature, two doses of the Sinopharm vaccine showed varying levels of short-term efficacy against symptomatic COVID-19 6 months after vaccination, with estimates of efficacy and effectiveness ranging from 83–5% in Turkey, 50–7% in Brazil, and 65–9% in Chile. Efficacy against hospitalizations due to COVID-19 hospitalizations was higher at 83-7% (95% CI 58–0–93–7) in Brazil and 87–5% (86–7 to 88–2) in Chile [16,17,18,19]. Phase 3 of randomized clinical trials of inactivated vaccine showed an efficacy of 50.7% and higher effectiveness against severe diseases [5,20]. We have also previously shown that the primary vaccine produced by Sinopharm showed moderate efficacy against symptomatic infections and good efficacy against COVID-19 [13].

During the 6-month follow-up period, despite a gradual decline in vaccine efficacy, Pfizer demonstrated a favorable safety profile and was highly effective in preventing COVID-19 [11,17,18]. The efficacy of the Pfizer vaccine against COVID-19 was 91.3% during the 6-month follow-up period in participants with no evidence of previous SARS-CoV-2 infection. The efficacy of the vaccine gradually decreased. Vaccine efficacy ranged from 86 to 100% across countries and populations of different ages, genders, ethnicities, and risk factors for COVID-19 in participants with no evidence of previous SARS-CoV-2 infection. The efficacy of the vaccine against the severe form of the disease was 96.7% [11,18].

In this study, many people vaccinated with inactivated vaccines who had no previous infection with SARS-CoV-2 had no or only low antibody levels three months after the basic immunization and before the booster vaccination. The results differ from those of people vaccinated with the Pfizer vaccine. In the group of people who received a primary series of the Pfizer vaccine, the anti-S1 IgG antibody levels showed a non-significant increase six months after the second dose and a non-significant decrease before the third vaccination dose. The situation with the Sputnik vaccine was similar to that with the Sinopharm vaccine; the values were very low until the third month after the second dose. After 6 months, there was an insignificant increase and then an insignificant decrease before the third dose was administered. We were also able to show that previous infection with SARS-CoV-2 in the interim, between the initial vaccination and the booster dose or after the booster, altered the immune response by increasing the anti-S1 antibody titer in all vaccinated individuals, regardless of the type of vaccine.

The antigen-specific IgG antibodies formed during a natural infection are usually long-lived and persist forever in the event of repeated infection. In the case of natural infection with SARS-CoV-2, however, the titer of specific IgG antibodies is high for up to three months and then decreases over time. Several studies reported rapid production of SARS-CoV-2-specific IgG within the first week of the infection, and most subjects had antibodies within 3 to 4 weeks of the disease onset [14,17]. Limited studies showed that antibody titers might be associated with older age, male gender, ethnicity, obesity, smoking, alcohol consumption, some underlying diseases, and the use of immunosuppressive drugs. The time of detection, the peak, and the duration of the antibodies also varied in the studies.

On the other hand, vaccine-induced immunity has its ups and downs. It decreases over time, and an additional dose of the vaccine is required for protection. In the meantime, antigenic drift of SARS-CoV-2 occurs, so a higher level of anti-S1 antibodies is required for protection against infection, especially in the context of new variants of concern (VOCs). The decrease in antibody levels also led to some loss of protection against hospitalization and death, especially in older adults [21]. For this reason, booster vaccination was recommended in our country, and personal choice of booster vaccine was available for all primarily vaccinated individuals.

In this study, we have shown that a third homologous booster vaccination with all tested vaccines significantly increases antibody responses compared to two doses of the primary series when administered approximately six months after the second dose. The individuals who received all three doses of the Pfizer or Sputnik V vaccines and had no history of COVID-19 during the observation period showed a statistically significant increase in anti-S1 IgG antibody levels one month after the third dose. Anti-S1 IgG antibody levels in people who had received all three doses of the Sinopharm vaccines without a history of COVID-19 were statistically significantly higher six months after the third dose. This result is consistent with our previous findings and data from the literature showing that a third dose of Sinopharm vaccine, a booster dose given 6 months after the second dose, results in a statistically significant increase in neutralizing antibody titers from a low baseline. Regarding the cellular immune response, a significant increase in IFN gamma was also reported in our previous article [13].

Furthermore, we analyzed the differences in the quality of humoral immunity after homologous and heterologous booster doses in individuals who had received an inactivated Sinopharm vaccine as their primary vaccination series. The heterologous regimen included the booster with the Pfizer vaccine. The results show that both the heterologous and homologous booster vaccination significantly increased the level of anti-S1 antibodies, with the heterologous booster vaccination being more potent than the homologous vaccination.

The very low titer of anti-S1 antibodies was detected 3 months after two doses of the inactivated vaccine, but both homologous and heterologous COVID-19 booster vaccinations strongly enhanced humoral immune responses. The literature also shows that the magnitude of the immune boost was more significant with the Sputnik V vaccine and the mRNA vaccine (Pfizer) than the homologous regimen, with the highest responses occurring after an mRNA boost [12,22,23]. In older adults, the difference in neutralizing antibody titers was 8–22-fold higher with a heterologous booster than a homologous booster of inactivated vaccine [12]. Our data did not confirm that, probably because of the small sample of older participants. A third dose of inactivated vaccine administered 6 months after the second dose resulted in an approximately 20-fold increase in neutralizing antibody titers from a low baseline, higher than the 12-fold increase observed for anti-spike IgG. Differences in the studied population and laboratory tests may explain the discrepancy in the absolute booster response. The booster doses with the viral vector and mRNA vaccines significantly increased the neutralizing capacity of serum samples for both the Delta and Omicron variants (at least 90% were seropositive after the booster). However, lower responses were observed after a Sinopharm booster, with only 35% becoming seropositive against Omicron [24,25]. Similarly, one article shows a 1.4-fold increase in neutralizing capacity against Omicron after an mRNA boost following two doses of the Sinopharm vaccine, compared to the activity of sera after two doses of the mRNA vaccine [11,24,25].

According to our studies, vaccine efficacy following basic immunization with two doses was observed for all vaccines used, as confirmed by the occurrence of SARS-CoV-2 infection by PCR results. Surprisingly, the mRNA-based vaccine (Pfizer) and the recombinant RNA vaccine (Sputnik V) demonstrated slightly lower efficacy in our study group six months after full immunization than the inactivated Sinopharm vaccine, which contradicts previously published data [26]. Interestingly, when efficacy is assessed solely by detecting anti-S1 antibodies, the results are reversed: the anti-S1 antibody titer is lowest in recipients of the Sinopharm vaccine and highest in those who received the Pfizer vaccine, aligning with the literature. The booster dose significantly increased antibody titers, particularly in individuals who received a heterologous booster. However, vaccine effectiveness waned over time, as evidenced by PCR-positive findings. This decline cannot be attributed to reduced anti-S1 antibody titers, which remained high even six months post-booster. Instead, it is likely due to the dominance of the Omicron variant during the post-booster period. The highest vaccine efficacy was observed before the booster dose, with effectiveness against the Alpha and Beta variants ranging from 14% to 26%. The best outcomes were noted in individuals who received three doses of the Sinopharm vaccine. Efficacy was determined based on PCR-confirmed findings before the booster dose, revealing that 14% of individuals vaccinated with Sinopharm were infected, compared to 24% for Pfizer, 26% for Sputnik, or 18% for a combination of Sinopharm and Pfizer. However, vaccine efficacy declined over time, likely due to Variants of Concern (VOCs) dominance shifts. Notably, primary vaccinations were administered during the dominance of the Alpha variant, while booster doses were given during the dominance of the Omicron variant. Thus, it is not surprising that efficacy decreased after the booster dose. The efficacy, determined by PCR-confirmed findings after the booster dose, was 70% for individuals boosted with Sinopharm, compared to 76% for those with Pfizer, 78% for Sputnik, or 82% for individuals primarily vaccinated with Sinopharm and boosted with Pfizer (Table 2).

Despite the benefits of vaccination, there is increasing evidence that reduced vaccine efficacy (attenuated humoral immunity) may be associated with repeated vaccination [27]. Recent publications have addressed the mechanisms underlying this deficiency. These generally relate to either a viral antigen clearance model, in which pre-existing antibodies lead to rapid clearance of vaccine antigens, or an FcR-mediated B cell negative signaling event, in which circulating antibodies bind to vaccine components and inhibit B cell activation, and finally, an epitope masking model in which circulating antibodies bind and mask specific epitopes of neutralizing epitopes while allowing stimulation of B cells with specificity for more distant epitopes [28,29].

In our study, the third dose of Pfizer and Sputnik V vaccines significantly improved the humoral immune response approximately one month after the third dose with significantly higher levels of antibodies than approximately one month after the second vaccination. This was no different in people primarily vaccinated with the Sinopharm vaccine and boosted with the third dose of Pfizer. However, in people vaccinated and had COVID-19 before the third dose, the increase in anti-S1 antibodies after the booster is insignificant. These results suggest that increasing antibody titer in individuals with previous infection and three doses of the vaccine is challenging. It also showed that the rate of decline in specific SARS-CoV-2 titers after the third dose of the vaccine was significantly slower than after the second dose.

However, the mutation and selection of new variants of concern necessitate regular immunization with vaccines containing the updated protein variant. As SARS-CoV-2 continues to circulate and antigenic drift persists, new variants occasionally emerge against which vaccine-induced immunity proves insufficient [30,31,32,33]. For instance, the Omicron variant of SARS-CoV-2, characterized by increased transmissibility, has been classified as a variant of concern. The Omicron variant features over 37 amino acid substitutions in the spike protein, 15 located in the receptor-binding domain (RBD). It has been predicted that some of these substitutions facilitate the evasion of neutralizing antibodies, critical determinants of protection against infection [20,34,35]. As we observed an increase in PCR-positive cases over time after the booster dose (Table 3), it suggests that annual vaccination might be advisable for immunocompromised individuals, similar to influenza immunization practices, due to their increased risk of severe illness and the potential for waning immunity over time [36]. Consequently, our future research will focus on investigating this phenomenon further.

## 4. Materials and Methods

### 4.1. Study Design

This prospective longitudinal cohort study assessed the dynamics of the humoral immune response in immunized people after administering the second and third doses of the SARS-CoV-2 vaccine. The humoral immune response was measured three and six months after the second dose of the SARS-CoV-2 vaccine; before the third dose of the SARS-CoV-2 vaccine; and one, three, and six months after the third dose of the SARS-CoV-2 vaccine. All participants included in the study voluntarily signed an informed consent form, completed a self-questionnaire (Appendix A), and had their blood drawn to perform the testing. The study was approved by the Institutional Ethics Committee of the Institute for Biocides and Medical Ecology, Belgrade (protocol number 05-01 1575/4-2, approved on 18 June 2021). The inclusion criteria were (I) older than 18 years; (II) previous vaccination with two doses of SARS-CoV-2 vaccines; (III) vaccination with a booster (third) dose of SARS-CoV-2 vaccines; and (IV) signing of informed consent. The exclusion criteria for our study were (I) younger than 18 years and (II) not vaccinated against SARS-CoV-2. A previous infection with SARS-CoV-2 was not an exclusion criterion.

### 4.2. Participant Selection and Serum Collection

All participants who met the inclusion criteria and provided informed consent were included in the study and categorized into groups according to the type of third dose of SARS-CoV-2 vaccines, gender, history of natural SARS-CoV-2 infection, and the presence of cardiovascular, pulmonary, autoimmune, endocrine, nervous, liver, and kidney diseases.

Serum samples were obtained by co-llecting 4 to 6 mL of whole blood in VACUETTE^®^ Serum Tubes (Greiner Bio-One GmbH, Kremsmünster, Austria). The blood was centrifuged at 3000 rpm for 10 min (Gyrozen model 416 centrifuge) before aliquoting the serum. Upon testing, the serum was stored at −20 °C. All the samples were collected between August 2021 and June 2022.

The study included a total of 410 individuals, 178 males and 232 females. Anamnestic data for the participants is presented in Table 1. Of the 410 individuals, 156 received all three doses of Sinopharm [Vero Cell]-Inactivated COVID-19 vaccine, while 88 received all three doses of Pfizer-BioNTech COVID-19 Vaccine; 33 received all three doses of the Sputnik V vaccine, and 133 received the first and second doses of the Sinopharm [Vero Cell]-Inactivated COVID-19 vaccine, as well as the third dose of the Pfizer-BioNTech COVID-19 vaccine.

**Table 1:** Anamnestic data for participants in the study.

Anamnestic Data				
Number of participants				
Total number	410		Age	
All three doses of the Pfizer-BioNTech COVID-19 Vaccine	88		49 ± 2	
All three doses of the Sinopharm [Vero Cell]-Inactivated COVID-19 vaccine	156		54 ± 1	
All three doses of the Sputnik V Vaccine	33		53 ± 2	
1st and 2nd dose of the Sinopharm [Vero Cell]-Inactivated COVID-19 vaccine, 3rd dose of the Pfizer-BioNTech COVID-19 Vaccine	133		52 ± 1	
Sex	W	M		
	232	178		
Presence of cardiovascular diseases	Yes	110	Hypertension (*n* = 91)	
			Pericarditis (*n* = 2)	
			Heart valve diseases (*n* = 4)	
			Other cardiovascular diseases (*n* = 13)	
	No	300		
Presence of diseases of the nervous system	Yes	9	Stroke (*n* = 3)	
			Epilepsy (*n* = 2)	
			Other diseases of the nervous system (*n* = 4)	
	No	401		
Presence of endocrine diseases	Yes	38	Diabetes mellitus (*n* = 19)	
			Thyroid gland diseases (*n* = 14)	
			Other diseases of the endocrine system (*n* = 5)	
	No	372		
Presence of liver diseases	Yes	5		
	No	405		
Presence of kidney diseases	Yes	3		
	No	407		
Presence of pulmonary diseases	Yes	13		
	No	397		
Presence of allergic reactions	Yes	51		
	No	359		
Presence of autoimmune diseases	Yes	10		
	No	400		

All participants were divided according to the history of previous SARS-CoV-2 infections. Based on the date of the last positive PCR result, out of 88 participants who received all three doses of the Pfizer-BioNTech COVID-19 Vaccine, 33 had positive PCR results. Among them, 8 (24%) had positive PCR results before a third dose of SARS-CoV-2 vaccine (Table 2). In the case of participants who received all three doses of Sinopharm [Vero Cell]-Inactivated COVID-19 vaccine, 14 out of 100 (14%) had positive PCR results before a third dose of Sinopharm [Vero Cell]-Inactivated COVID-19 vaccine.

**Table 2:** History of SARS-CoV-2 infection in participants.

		Number of Participants						
	Total		SARS-CoV-2 History	Most Recent PCR-Positive Results			% PCR Positive	
		Yes	No	Before 3rd Dose	After 3rd Dose	Before and After 3rd Dose	Before 3rd Dose	After 3rd Dose
All three doses of the Pfizer-BioNTech COVID-19 vaccine	88	33	55	8	25	0	24%	76%
All three doses of the Sinopharm [Vero Cell]-Inactivated COVID-19 vaccine	156	100	56	14	70	16	14%	70%
All three doses of the Sputnik V vaccine	33	19	14	5	11	3	26%	78%
1st and 2nd dose of the Sinopharm [Vero Cell]-Inactivated COVID-19 vaccine, 3rd dose of the Pfizer-BioNTech COVID-19 vaccine	133	61	72	11	50	0	18%	82%

Participants with the most recent PCR positive results following the third dose of the SARS-CoV-2 vaccination were categorized into four groups:

- The first group comprised those whose most recent PCR-positive results were obtained within one month following the third dose of the SARS-CoV-2 vaccine.
- The second group consisted of those with PCR-positive results from one to three months after the third dose of the SARS-CoV-2 vaccine.
- The third group consisted of those with PCR-positive results from three to six months after the third dose of the SARS-CoV-2 vaccine.
- The fourth group had PCR-positive results more than six months after the third dose of the SARS-CoV-2 vaccine.

Based on the above-mentioned criteria, of the 70 participants who received all three doses of the Sinopharm [Vero Cell]-Inactivated COVID-19 vaccine, 12 had their last positive PCR results one month after the third dose, 15 had theirs three months after the third dose, 17 of them had theirs six months after, and 26 had their last positive PCR results more than six months after the third dose of SARS-CoV-2 vaccine (Table 3).

**Table 3:** Participants distribution based on PCR results.

	Number of Participants			
	1 Month 3rd Dose	3 Months 3rd Dose	6 Months 3rd Dose	More Than 6 Months 3rd Dose
All three doses of the Pfizer-BioNTech COVID-19 Vaccine	4	7	6	8
All three doses of the Sinopharm	12	15	17	26
All three doses of the Sputnik V Vaccine	2	3	5	4
1st and 2nd dose of the Sinopharm, 3rd dose Pfizer-BioNTech COVID-19 Vaccine	3	10	19	18

Of 410 participants, 110 had cardiovascular diseases, 38 had endocrine diseases, 51 had allergic reactions, and 13 had lung diseases (Table 1). None of them were pregnant, breastfeeding, or had primary and secondary immuno-deficiencies or diseases of the hematopoietic system. A total of 91 of the 110 individuals with cardiovascular diseases had hypertension, 2 had pericarditis, 4 had heart valve diseases, and 13 had other cardiovascular diseases (Table 1). In the case of the presence of endocrine diseases, nineteen had diabetes mellitus, and fourteen of them had thyroid gland diseases.

### 4.3. SARS-CoV-2 Serological Analyses

To detect the humoral immune response against SARS-CoV-2, we used commercially available Anti-SARS-CoV-2 QuantiVac ELISA (IgG) (EUROIMMUN AG, Lübeck, Germany. According to the manufacturer’s instructions, all serological analyses were performed on the EUROIMMUN Analyzer I platform. The Anti-SARS-CoV-2 QuantiVac ELISA (IgG) assay is based on 96-well microplates coated with the SARS-CoV-2 S1 domain (including RBD) expressed recombinantly in the human cell line HEK293 (ATCC). Quantification of S1-specific IgG was performed using a 6-point calibration curve covering a range from 1 to 120 relative units (RU)/mL. Results < 8 RU/mL were considered negative, 8–11 RU/mL as borderline, and ≥11 RU/mL as positive. The lower detection limit for undiluted samples was <1.2 RU/mL, and the upper detection limit was >120 RU/mL. Positive and negative controls were included in each test run. The sensitivity of this test, when used at 10 days and 21 days after symptom onset, was 90.3% and 93.2%, respectively. The specificity of this ELISA test amounted to 99.8%.

### 4.4. Statistical Analysis

Statistical analyses were performed according to protocols described by Hinkle et al. [37]. The groups were analyzed using analyses of variance (ANOVA), with post hoc comparisons by Tukey’s HSD *t*-test; a *p* < 0.05 was considered significant. Correlation analysis was performed using Pearson’s correlation protocol.

## 5. Conclusions

Like other authors, we demonstrated that three doses of the Pfizer, Sputnik V, or Sinopharm vaccines produce good anti-S1 antibodies. However, this antibody production level is insufficient to protect against new VOCs of SARS-CoV-2, such as the Omicron variant, even six weeks after booster vaccination. Homologous or heterologous booster doses with the Pfizer vaccine following two doses of Pfizer or Sinopharm enhance anti-S1 antibody levels. Three doses of the Sinopharm vaccine also elicit strong antibody responses in most recipients. However, the antibody level is not always directly correlated with protective immunity. It can be assumed that Sinopharm elicits a broader range of virus-specific T-cell responses, which may help to compensate for some loss of antibody protection.

## Figures and Tables

**Figure 4:**
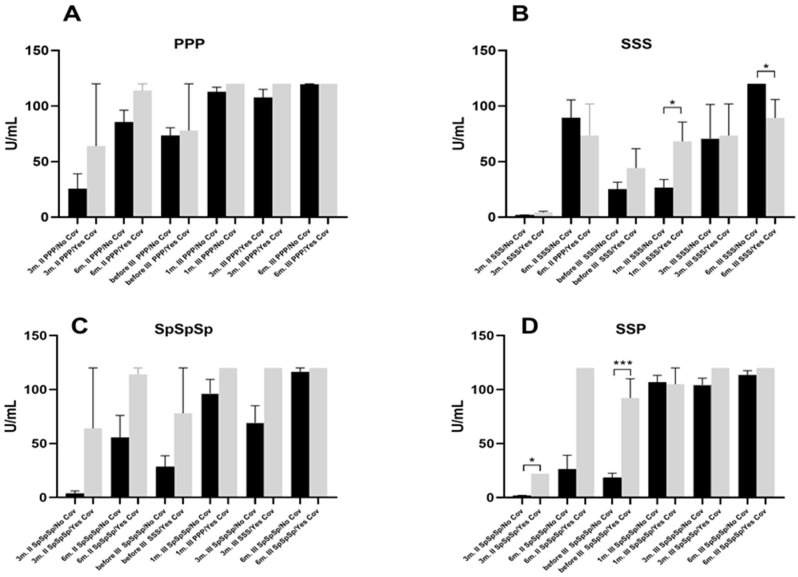
Comparison of anti-S1 IgG antibody levels in individuals with history/no/of COVID-19 in different time points 3 and 6 months after the second (II) dose; before the third (III) dose; and 1, 3 and 6 months after the third dose during vaccination with all three doses of Pfizer (PPP) (**A**); all three doses of Sinopharm (SSS) (**B**); all three doses of Sputnik vaccine (SpSpSp) (**C**); and a combination of the Sinopharm and Pfizer vaccine (two doses of Sinopharm and third dose of Pfizer, SSP) (**D**). * *p* < 0.05; *** *p* < 0.001.

## Data Availability

Data are not available due to ethical restrictions.

## Appendix A

Participant self-questionnaire:

All participants who gave their informed consent voluntarily filled out a self-questionnaire with the following inquiries:

- History of previous SARS-CoV-2 infections with the date on which the symptoms appeared or the date of the last positive PCR result;
- History of vaccination (date and name of 1st, 2nd, and 3rd dose of the SARS-CoV-2 vaccine);
- Diseases of the cardiovascular system (hypertension, ischemic heart disease, heart failure, heart valve diseases, myocarditis, endocarditis, pericarditis, deep vein thrombosis, etc.);
- Diseases of the endocrine system (diabetes mellitus, metabolic syndrome, hyperthyroidism, hypothyroidism, Cushing’s syndrome, etc.);
- Diseases of the nervous system (cerebrovascular diseases, stroke, epilepsy, multiple sclerosis, polyneuropathy, neuroborreliosis, etc.);
- Liver diseases (hepatitis B, hepatitis C, cirrhosis, etc.);
- Autoimmune diseases (systemic lupus, rheumatoid arthritis, systemic sclerosis, etc.);
- Pulmonary diseases (asthma, COPD, emphysema, pulmonary hypertension, etc.);
- Kidney diseases (hypertensive nephropathy, diabetic nephropathy, hydronephrosis, chronic renal insufficiency, etc.);
- The presence of allergic reactions (atopy, allergic dermatitis, allergic rhinitis, allergic asthma, etc.);
- Primary and secondary immunodeficiencies (yes or no, and which);
- Severe diseases of the hematopoietic system (yes or no, and which);
- Oncological diseases (yes or no, and which);
- Pregnancy and breastfeeding status.
